# Genetic Diversity in the UV Sex Chromosomes of the Brown Alga *Ectocarpus*

**DOI:** 10.3390/genes9060286

**Published:** 2018-06-06

**Authors:** Komlan Avia, Agnieszka P. Lipinska, Laure Mignerot, Alejandro E. Montecinos, Mahwash Jamy, Sophia Ahmed, Myriam Valero, Akira F. Peters, J. Mark Cock, Denis Roze, Susana M. Coelho

**Affiliations:** 1 Sorbonne Université, UPMC Univ Paris 06, CNRS, Algal Genetics Group, Integrative Biology of Marine Models, Station Biologique de Roscoff, CS 90074, 29688 Roscoff, France; alipinska@sb-roscoff.fr (A.P.L.); laure.mignerot@sb-roscoff.fr (L.M.); mahwash.jamy@ebc.uu.se (M.J.); S.M.Ahmed@leeds.ac.uk (S.A.); cock@sb-roscoff.fr (J.M.C.); coelho@sb-roscoff.fr (S.M.C.); 2 Evolutionary Biology and Ecology of Algae, CNRS, Sorbonne Universités, UPMC, University of Paris VI, UC, UACH, UMI 3614, 29688 Roscoff, France; jano.montecinos@gmail.com (A.E.M.); myriam.valero@sb-roscoff.fr (M.V.); denis.roze@sb-roscoff.fr (D.R.); 3 Facultad de Ciencias, Instituto de Ciencias Ambientales y Evolutivas, Universidad Austral de Chile, Casilla 567, Valdivia, Chile; 4 Bezhin Rosko, 29250 Santec, France; akirapeters@gmail.com

**Keywords:** UV sex chromosomes, pseudoautosomal region, brown algae, neutral diversity

## Abstract

Three types of sex chromosome system exist in nature: diploid XY and ZW systems and haploid UV systems. For many years, research has focused exclusively on XY and ZW systems, leaving UV chromosomes and haploid sex determination largely neglected. Here, we perform a detailed analysis of DNA sequence neutral diversity levels across the U and V sex chromosomes of the model brown alga *Ectocarpus* using a large population dataset. We show that the U and V non-recombining regions of the sex chromosomes (SDR) exhibit about half as much neutral diversity as the autosomes. This difference is consistent with the reduced effective population size of these regions compared with the rest of the genome, suggesting that the influence of additional factors such as background selection or selective sweeps is minimal. The pseudoautosomal region (PAR) of this UV system, in contrast, exhibited surprisingly high neutral diversity and there were several indications that genes in this region may be under balancing selection. The PAR of *Ectocarpus* is known to exhibit unusual genomic features and our results lay the foundation for further work aimed at understanding whether, and to what extent, these structural features underlie the high level of genetic diversity. Overall, this study fills a gap between available information on genetic diversity in XY/ZW systems and UV systems and significantly contributes to advancing our knowledge of the evolution of UV sex chromosomes.

## 1. Introduction

Morphologically distinct sex chromosomes have evolved multiple times independently in both plants and animals [1]. Sex chromosome evolution has been mainly studied in male-heterogametic (XX/XY) and female heterogametic (ZZ/ZW) sex determination systems. A typical sex chromosome pair derives from a pair of autosomes through the acquisition of genes involved in sex determination. If more than one locus involved in sex determination is located on the chromosome, recombination between these loci is expected to be suppressed, leading to the establishment of a non-recombining region on the nascent sex chromosome, the sex-determining region or SDR. The formation of this non-recombining region has important consequences for the evolution of this part of the genome. Repetitive DNA can accumulate, leading to an increase in SDR size (see review by Bachtrog [1]). There is also a tendency for genes within the SDR to degenerate as a consequence either of an accumulation of deleterious mutations or of a lower rate of adaptation [1,2]. At a later stage, deletion of non-functional DNA from within the SDR may lead to a decrease in the size of the SDR. Furthermore, the SDR can progressively expand into the flanking regions of the chromosome (the pseudoautosomal regions, PAR), so that it encompasses an increasingly greater proportion of the sex chromosome.

Evolutionary processes at a given site in a genome are influenced by selection acting on closely linked sites, an effect called Hill-Robertson interference [3,4]. This selection interference reduces the effective population size (N_e_) experienced by the site in question [5]. As this effect is expected to be maximal in regions experiencing little or no recombination, diversity in SDRs such as Y-linked regions will be reduced compared to that of autosomes or pseudo-autosomal regions. In addition, loci on the Y chromosome are expected to experience a N_e_ that is one-quarter that of autosomal, and one-third that of X-linked genes. Moreover, since the level of neutral polymorphism maintained at equilibrium is proportional to the product of N_e_ and the neutral mutation rate, µ (π = 4Neµ) [6], diversity should be lower for Y-linked genes than for their X-linked counterparts and diversity in both X and Y genes should be lower than for autosomal genes [7]. Note, however, that this does not apply to genes located in the PAR, which should have the same N_e_ as autosomal genes. Accordingly, in *Silene latifolia* for example, diversity has been shown to be reduced in the Y-linked regions relative to X-linked regions [8]. A similar situation was observed in *Drosophila* [9] and in Saudi-Arabian hamadryas baboons [10].

In contrast to the SDR, the PARs of sex chromosomes maintain similarity between alleles of the same gene because they undergo homologous pairing and recombination. Therefore, genes in the PARs are expected to evolve in a similar manner to autosomal genes [11,12]. However, because of the proximity to the SDR, PARs are expected to display specific evolutionary dynamics [13]. One of these specificities is that linkage with the SDR widens the conditions allowing the maintenance of polymorphism at loci under sexually antagonistic selection and also increases neutral diversity due to longer coalescence times [14,15,16]. Diversity in PARs is therefore predicted to be high in those regions in close proximity with the SDR [17]. Increased genetic diversity, and overall footprints of balancing selection due to sexually-antagonistic selection, have been observed for several *Silene latifolia* PAR loci, although there was no evidence for an effect of proximity to the SDR [18,19].

**Figure 1:**
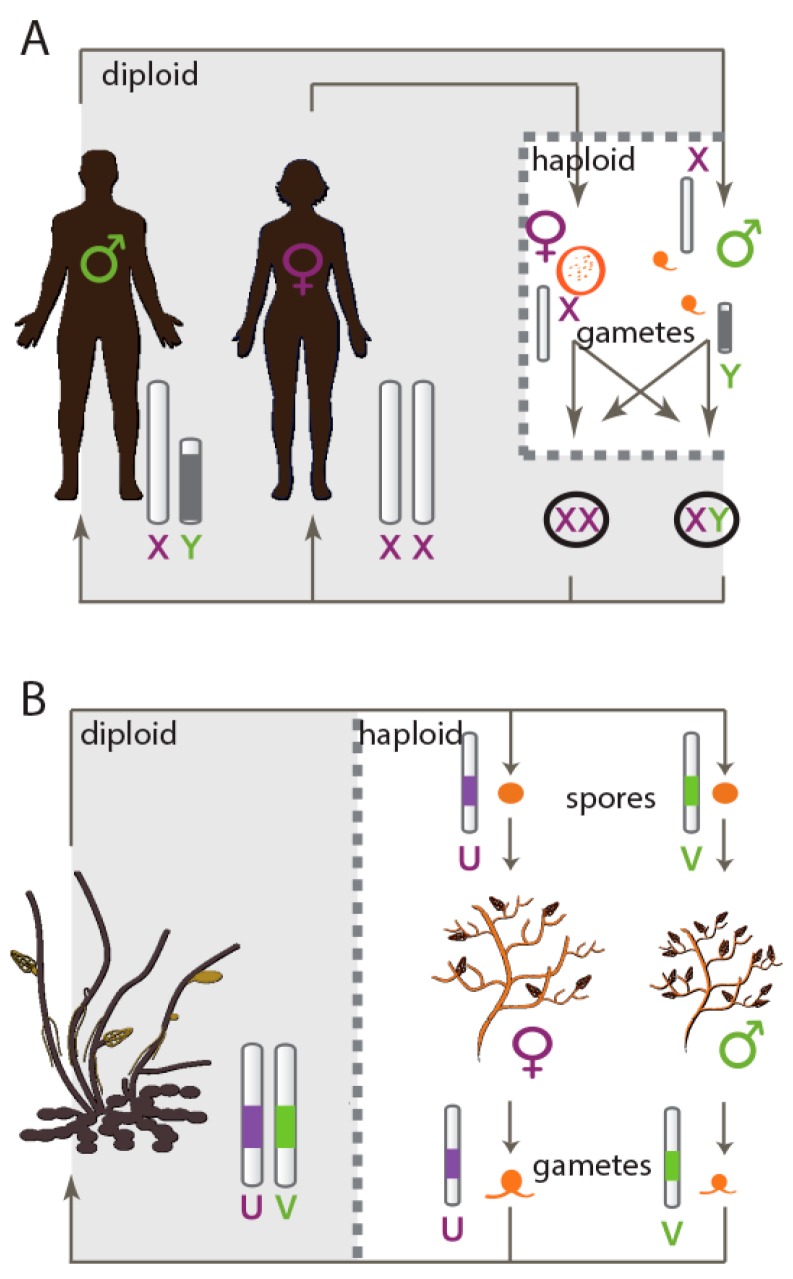
Comparisons of XX/XY and UV sex determination systems. (**A**) XX/XY sex determination system. The sexual individuals are diploid, and the sex of an offspring is determined after fertilization, depending on the sex chromosome contributed by the sperm. Please note that the haploid phase of the life cycle is limited to the gametic stage. Note also that ZW systems function in a similar manner to XY systems, with diploid phase sex determination, but it is the female that is the heterogametic sex; (**B**) UV sex determination system. The diploid, asexual generation (sporophyte) carries both the U and the V sex chromosomes which are passed on to the haploid spores after meiosis. Spores that receive the V sex chromosome develop into a male gametophyte whereas spores carrying U sex chromosome will produce a female gametophyte. Egg and sperm produced by the gametophyte fuse to return to the diploid generation. In UV sex determination systems, the sexual individuals are haploid and sex chromosomes function in the haploid state.

While information (theoretical and empirical) is available for XY and ZW sex chromosome systems, we know very little about evolutionary process, and in particular about patterns of genetic diversity, in a third type of sexual system that exists in nature, UV sex chromosomes (see review by Wilson Sayres [20]). In UV systems, which are very common in non-vascular plants and red, green and brown algae, sexes are expressed during the haploid stage of the life cycle, and females carry a U chromosome whereas males carry a V chromosome [21,22,23]. UV sexual systems (Figure 1) have specific evolutionary and genetic properties, including the absence of homozygous or heterozygous sexes and the absence of masking of deleterious mutations during the haploid phase when sex is expressed. Another significant feature that distinguishes UV from diploid sex chromosomes is that the N_e_ for U- and V-specific regions is expected to be half that of autosomes [21] and both the U and the V are theoretically subject to the same mutation rate (unlike XY or ZW systems where, for example, the Y can have a higher mutation rate than the X [24,25]). The PAR region is expected to have the same N_e_ as autosomes.

The only detailed studies focusing on the structure and evolution of both the SDR and PAR regions of UV systems have been carried out in the brown algal model organism *Ectocarpus* sp. [26,27,28]. In this organism, the U and V-specific regions are small, and exhibit mild degeneration despite the action of haploid purifying selection [26]. SDR genes were shown to evolve rapidly, mainly due to relaxed purifying selection [29]. Remarkably, the relatively large *Ectocarpus* PARs exhibit unique features. Although they recombine normally, these regions differ from autosomes in terms of their gene density, transposable element content and genetic structure [28]. Moreover, the PAR is significantly enriched, compared to autosomes, in genes expressed specifically or predominantly during the diploid, sporophyte phase of the life cycle (hereinafter called sporophyte-biased or SP-biased genes), and these genes have been shown to evolve faster than unbiased genes [28]. A model was proposed to explain this enrichment phenomenon, giving SP-biased genes an advantage to spread when they were partially linked to the SDR and had a positive effect on fitness in one of the sexes [28]. The model assumes that the evolution of the PAR in haploid systems is under the influence of differential selection pressures in males and females acting on alleles that are advantageous during the sporophyte generation of the life cycle.

Here we used extensive Double Digest Restriction Associated DNA sequencing (ddRAD-seq) data combined with a gene-by-gene approach to perform a comprehensive analysis of the genetic diversity across the UV sex chromosome of the brown alga *Ectocarpus*. We show that the level of neutral diversity in the U and V SDR haplotypes is about half that of the autosomes. This observation is in line with theoretical predictions based on the reduced effective population size of the SDR (the U and V SDR each have half the N_e_ of the autosomes), suggesting that the influence of additional factors such as background selection or selective sweeps is minimal. Interestingly, genetic diversity in the PAR region was surprisingly elevated and there were several indications that genes in this region may be under balancing selection.

## 2. Materials and Methods

### 2.1. Quantitative Trait Loci (QTL) Mapping of the Sex Locus in Ectocarpus Siliculosus

The populations analyzed in this study belong to the species *Ectocarpus siliculosus*, a sister species to *Ectocarpus* sp. for which a reference genome sequence and genetic map with detailed coordinates of the positions of the SDR and pseudoautosomal regions are available [27,30,31,32]. The *Ectocarpus* sp. reference genome strain still lacks a formal species name. It was referred to as *Ectocarpus* 7 in a recent phylogenetic analysis paper [33] and we therefore also referred to it as *Ectocarpus* 7 throughout this paper. To confirm that the borders of the SDR were the same in the two species, in the absence of a complete genome sequence for *E. siliculosus*, we generated a genetic map for this species [34] focusing specifically on the sex chromosome, to investigate the location of the SDR, as described below.

A diploid sporophyte (Ec236) was generated by crossing two compatible *E. siliculosus* strains from a Naples population (EA1 and RB1) (Table S1). From this sporophyte, 152 haploid gametophytes were isolated, each arising from a unique meiotic event [35,36]. The sex of each individual was determined using sex-specific PCR markers [37]. Molecular methods, ddRAD sequencing of this population and detailed analysis of the genetic map obtained are described in the Supplementary Materials and Methods.

#### QTL Mapping of the Sex Locus

The genetic map obtained with the 152 haploid gametophyte progeny derived from the diploid sporophyte Ec236 was used to map the *E. siliculosus* SDR. Using the sex of the progeny as a binary trait, the SDR location was determined as a QTL in the R package R/qtl [38] with the scanone function and the “binary” model. To confirm its position, we also used MapQTL [39] with the Kruskal-Wallis non-parametric method. The R/xoi package (version 0.67–4) [40] was used to obtain a smoothed estimate of the recombination rate along the linkage groups (LGs), in 1 Mbp sliding windows.

### 2.2. Sequencing of the Individuals from Different Natural Populations for Population Genomics Analyses

#### 2.2.1. Field Sample Unialgal Collections

We selected several populations from different geographical origins in order to test the repeatability of the observed pattern of neutral variation between genomic regions in face of the population history or environment conditions of this cosmopolitan species. The samples used were previously obtained from natural populations collected along the European coast of the Atlantic and Mediterranean Sea and the Pacific coast of Chile by Couceiro, et al. [41] and Montecinos, et al. [42] (Table S2). Samples were maintained in the lab as unialgal cultures as described in Couceiro, et al. [41].

#### 2.2.2. Analyses of Neutral Diversity in Non-Recombining versus Recombining Regions

ddRAD-sequencing is a reduced-representation genome sequencing method that involves digestion of the genomic DNA with two different restriction enzymes followed by size selection, PCR amplification and sequencing of the obtained library on a sequencing platform such as Illumina MiSeq or HiSeq [43]. ddRAD-seq data were generated for 49 diploid *E. siliculosus* individuals (sporophytes), representing three European and one South American population and 6 additional haploid individuals (gametophytes) from two European and one South American population. Sample information and accession numbers are given in Table S2. Sequencing methods followed the protocols described in [30]. Sequences consisted of paired-end reads obtained using the Illumina HiSeq 2500 system (Illumina Inc., San Diego, CA, USA). Final reads were trimmed to 70 bp. After quality (QUAL >30 and minimum genotype quality =40) and missing data filtering (not more than 40% of missing data per sample and not more than 40% of missing data per locus), 39 samples were retained for further analysis.

Quality-filtered reads were mapped to the *Ectocarpus* 7 reference genome using BWA [44] with the parameters “bwa mem -M -c 2”. The genetic map of the reference *Ectocarpus* 7 genome strain provided high-quality annotation and allowed the compartmentalization of the mapped reads to autosomal, pseudoautosomal and non-recombining regions. Genotypes were called using samtools mpileup v.1.6 [45] and filtered using vcftools v.0.1.15 [46]. Only high-quality genotype calls (Phred-scaled mapping quality and genotype quality ≥20) were retained and sites with more than 25% missing data were excluded from the downstream analysis. Additionally, since coding sequences can experience positive or negative evolution that will affect their diversity patterns (see [47]), we excluded regions overlapping with exons to minimize the effect of selection and focused on neutrally evolving sites. We used samtools mpileup to report non-variant sites as well as polymorphic sites in order to concatenate the sequenced portion of the genome and assemble a “reduced” genomic sequence. 

The method described above was validated by employing an alternative approach, which is described in the Supplementary Materials. Briefly, this method was based on using the Stacks pipeline [48] to carry out a *de novo* analysis of the ddRAD-seq data. The main difference between the first method described above and this alternative method was that, in the latter, only bi-allelic variant positions were called with the Stacks pipeline. Because both approaches gave consistently similar outputs, we focus here on the results obtained using the first method.

#### 2.2.3. Analyses of Non-Recombining and Recombining Coding Regions

In addition to the ddRAD sequencing data, we used cDNA obtained from a total of 20 individuals (11 males and 9 females) from an *E. siliculosus* population from Naples (Italy) to provide further information about genetic diversity in the coding regions of autosomes and the sex chromosome (Table S3). Total RNA was extracted using a chloroform-isoamyl alcohol protocol (adapted from [45]). The SuperScript IV Reverse Transcription System kit (Thermo Fisher Scientific Inc., Villebon sur Yvette, France) was used to synthesize cDNA using random hexaprimers and oligo primers according to manufacturer’s instructions. Primers were designed using Primer3 (Table S4). The coding regions of two autosomal, six PAR and five SDR genes (three male-specific and two female-specific) (Table 1) were amplified and sequenced on a 3130xl-3 capillary sequencer (Applied Biosystems Inc., Station Biologique de Roscoff, France). Amplicon sequences were processed with CodonCode Aligner v5.1.5 (http://www.codoncode.com).

### 2.3. Data Analysis

We used vcftools [46] and the concatenated genomic sequences described above to calculate nucleotide diversity (π) and Tajima’s *D* for non-overlapping 1-kb windows across the different genome compartments (autosomes, PAR and SDR). Since the SDR regions of both the U and the V are rather small (ca. 1Mbp), the windowed π and Tajima’s *D* values calculated separately for the U and V SDRs were grouped subsequently and represented as one global (concatenated) SDR region to increase the statistical power of the downstream analyses. We performed the analysis of the *E. siliculosus* species (all populations pooled) as a whole and also separately for each population where indicated.

Nucleotide diversity and its partition between synonymous (*p*_S_) and non-synonymous (*p*_N_) mutations and synonymous (*D*_S_) and non-synonymous (*D*_N_) divergence rates for the coding regions in the population from Naples were calculated in DnaSP v5.10.01 [49] using the reference genome strain (*Ectocarpus* 7) as an outgroup. Coalescent simulations were performed with recombination (where applicable) under the standard neutral model with 10,000 iterations to obtain values and 95% confidence intervals of Tajima’s *D* and Fay and Wu’s *H*. The direction of selection (DoS) statistic [50] was calculated using the following formula:

DoS = *D*_N_/[*D*_N_ + *D*_S_] − *p*_N_/[*p*_N_ + *p*_S_]

We used the Wilcoxon test to compare the neutral diversity (π) and Tajima’s *D* values generated per window across different genomic regions. All statistical analyses were performed in RStudio (R version 3.3.2) with graphs produced using the R package ggplot2 [51].

### 2.4. Data Availability

Data availability and accession numbers are described in Table S2. All sequences have been deposited in the Sequence Read Archive (SRA) under the ID SRP149054 (BioProject ID: PRJNA473288).

## 3. Results

### 3.1. Identification of the PAR and SDR in the Sex Chromosome of E. siliculosus

*E. siliculosus* is a closely related species to the *Ectocarpus* 7 genome-sequenced strain, whose assembly is of very high quality [27,31]. Knowledge of the exact position of the borders of the SDR and PAR is particularly important in the context of this study because theoretical models predict increased neutral diversity at the borders of the SDR and PAR regions [13,17]. To conduct the population genetic tests on the different genomic compartments, we needed to confirm that sister species *E. siliculosus* and *Ectocarpus* 7 (reference genome strain) shared the same PAR-SDR boundary. Previous studies had shown that the SDR of *E. siliculosus* and *Ectocarpus* 7 contain exactly the same genes [29]; however, the analysis focused on genes and did not determine whether the borders of the SDR were the same in the two species. We therefore used a newly generated genetic map for *E. siliculosus* [34] focusing specifically on the sex chromosome, to investigate the location of the SDR (Figure S1). A sex-specific QTL peak was detected at 50.9 cM on the linkage group 2 (362 markers over 104.3 cM). Based on the mapping of *de novo* assembled tags of *E. siliculosus* onto the reference genome, the SDR boundaries (position 2,775,867 bp to position 3,674,342 bp on the chromosome 13 of the reference genome of *Ectocarpus* 7 [52]) were found to be located overall at the same positions in the two species (Figure S1).

Taken together these analyses confirmed that the position of the SDR of the sex chromosome is similar in *E. siliculosus* and *Ectocarpus* 7 (Figure S1). Therefore, we concluded that the ddRAD-seq reads from *E. siliculosus* populations can be assigned to the different genomic compartments based on the *Ectocarpus* 7 genome annotation.

### 3.2. ddRAD-seq Data

We used ddRAD-seq data generated from samples collected from four populations of *E. siliculosus* (Table S2), three from Europe (Ribadeo and Gandario, in Spain and Roscoff in France) and one from South America (Pan de Azucar, Chile) to assess the extent to which genetic diversity in the male and female SDR, PAR and autosomes differed in different genomic regions. 

Once demultiplexed and cleaned, the ddRAD sequencing generated between 14.6 and 28.9 million sequence reads per population (Table S2). Based on the number of uniquely mapped reads to the *Ectocarpus* 7 reference genome, we estimated that the data covered ca. 13% of the reference genome sequence. This proportion of the genome captured is in the range of typical proportions captured by Restriction Associated DNA (RAD) sequencing methods (e.g., [53]) and RAD data has been shown to provide useful information for analyses such as neutral diversity studies [54,55]. After applying stringent filtering (see material and methods), more than 2 million sites were scored across the genome for all individuals including 187,062 SNPs (Table S5).

### 3.3. DNA Neutral Diversity

**Figure 2:**
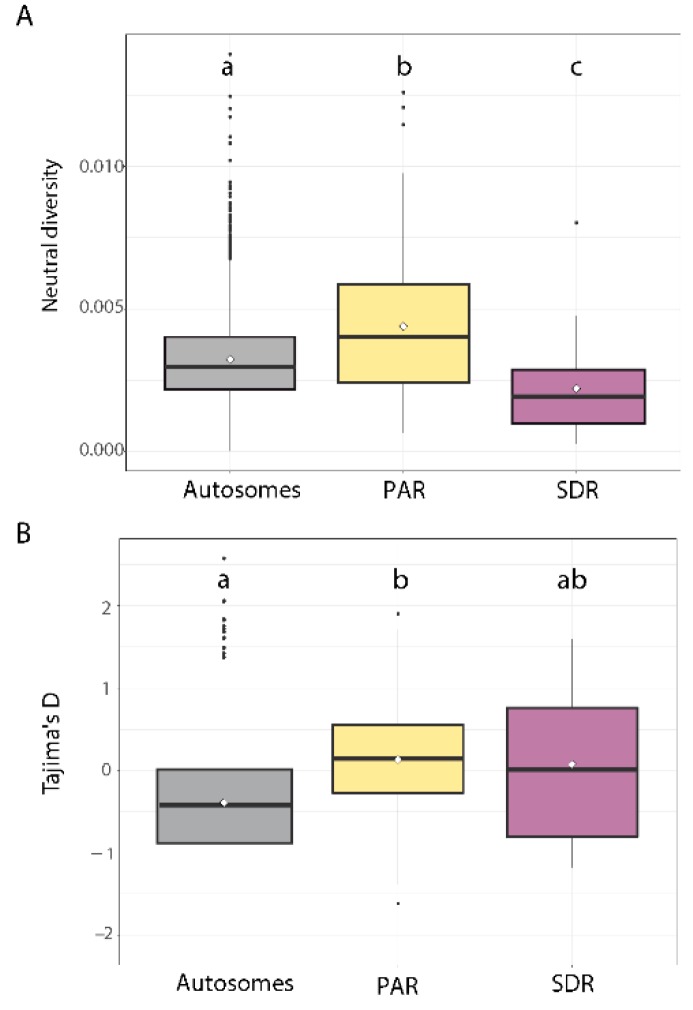
Population genetics statistics for the three *Ectocarpus siliculosus* genomic compartments: autosomes, pseudoautosomal regions (PAR) and sex-determining region (SDR). (**A**) Boxplots of neutral diversity (π), calculated in 1 kb windows without overlap; (**B**) Boxplots of Tajima’s *D*, calculated in 1 kb windows without overlap. The mean values are represented by the diamond shape. Letters above the boxplots denote significant differences (Wilcoxon test, *p* < 0.0005).

Sequence diversity was estimated for the autosomes, the PAR and the male and female SDRs (Figure 2A). The windowed values were calculated separately for the U and V SDRs (Figure S2) and grouped subsequently to be represented as one global SDR region. Neutral diversity (π) was relatively similar across all autosomes (mean π_A_ = 3.23 × 10^−3^ ± 3.47 × 10^−5^ SE) (Figure S3A). Genetic diversity on the SDR was approximately half that of the autosomes (mean π_SDR_ = 0.00221 ± 3.97 × 10^−4^ SE) and this difference was significant (Wilcoxon test, *p* = 0.0005) (Figure 2A), corresponding approximately to the equilibrium neutral expectations for the population size of these regions (the U and V SDR each have half of the N_e_ of the autosomes). Remarkably, however, the PAR exhibited significantly higher diversity (mean π_PAR_ = 4.39 × 10^−3^ ± 3.11 × 10^−4^ SE; Wilcoxon test, *p* = 0.0004) (Figure 2A and Figure S3A).

**Figure 3:**
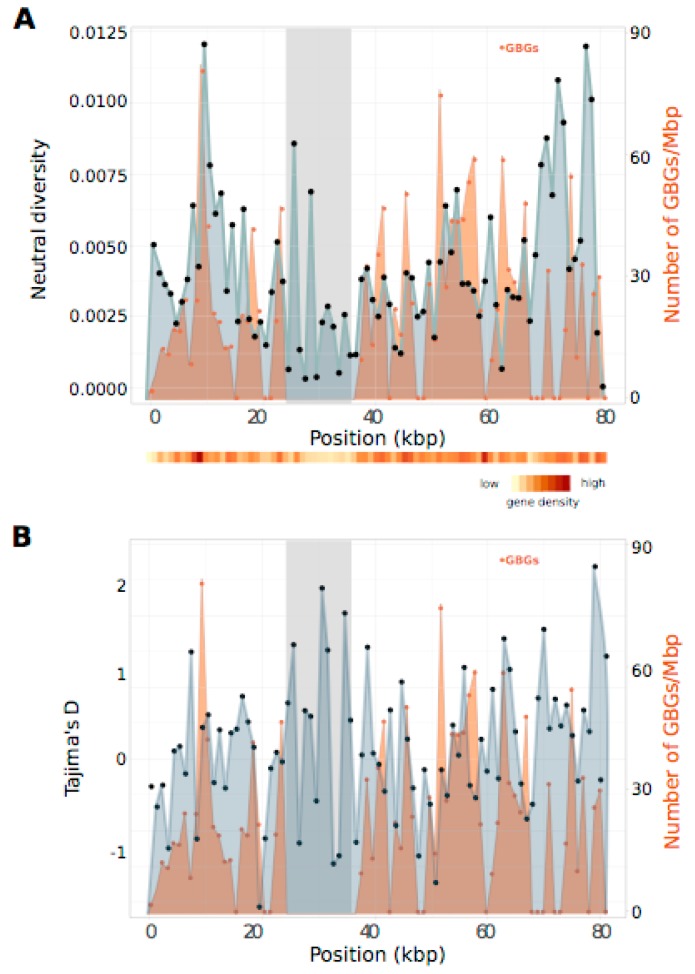
Population genetic statistics for the sex chromosome of *Ectocarpus siliculosus*. (**A**) Sliding window analysis of neutral diversity (π) in 1 kb non-overlapping windows along the concatenated ddRAD sequences of the sex chromosome. Values of π are indicated by black dots; (**B**) Sliding window analysis of Tajima’s *D* in 1 kb windows (indicated by black dots) along the concatenated sex chromosome sequence. The number of genes with differential expression in the sporophyte or gametophyte generation (generation-biased genes or GBGs) normalized by the physical distance covered by the concatenated 1 kb ddRAD window are marked in orange. Global gene density along the concatenated sex chromosome sequence is represented by the heatmap. The position of the sex-determining region is shaded in gray.

Although π values were higher overall in the PAR compared to the autosomes (Figure 2A), sliding window analysis along the sex chromosome (Figure 3A) did not show any clear bias towards elevated π regions being located close to the SDR. 

Another factor that could influence genetic diversity is gene density. Higher gene density implies more nearby sites potentially under selection and could influence the levels of diversity in the linked sites in a negative manner. Negative correlations between gene density and local levels of neutral diversity have recently been described in *Heliconius* species [47] We found a weak positive correlation between local gene density and neutral diversity along the PAR (Spearman’s rho = 0.27, *p* = 0.016), but no correlation (Spearman’s rho = 0.03, *p* = 0.915) for an autosomal chromosome of similar size (chr4). Positive correlation with gene density on the PAR could indicate increased polymorphism due to balancing selection. Recent work in *Silene* has shown that some of the loci on the PAR that exhibit high diversity may be under balancing selection [19], which is reflected by positive Tajima’s *D* values [56]. To test the possibility that the high diversity values found in the *Ectocarpus* PAR region reflect balancing selection we therefore performed Tajima’s *D* tests on the different genomic compartments (autosomes, PAR and SDRs) (Figure 2B and Figure 3B).

Overall Tajima’s *D* was negative for all autosomes (mean Tajima’s *D* = −0.389 ± 0.014 SE, Figure S3B). Strikingly, however, Tajima’s *D* showed positive values for the PAR that were significantly higher than values obtained for autosomes (mean Tajima’s *D* = 0.130 ± 0.090 SE, Wilcoxon test, *p* = 2.1 × 10^−8^) (Figure 2B). Tajima’s *D* was also elevated in the SDR (mean Tajima’s *D* = 0.0761 ± 0.196 SE) but was not significantly different from that of the autosomes.

### 3.4. Comparison of DNA Neutral Genetic Diversity Pattern between the Four Study Populations

The neutral diversity and Tajima’s *D* pattern along the sex chromosome of *E. siliculosus* was analyzed separately for each of the four study populations (Figure S4A,B respectively). The pattern of diversity in the PAR was similar despite their different geographical origin and diverse ecological environments (Figure S4A) (Wilcoxon pairwise test between populations, *p* > 0.2). The pattern of Tajima’s *D* on the other hand was more variable (Figure S4B), and this result may reflect differences among population history such as different demographic processes.

### 3.5. PAR Nucleotide Diversity and Generation-Biased Genes

The PAR of *Ectocarpus* exhibits unusual characteristics compared to the autosomes, including higher transposable element content, lower gene density and higher *D*_N_/*D*_S_ rates [28,57]. Of the 455 genes on the PAR, 177 show generation-biased expression patterns (out of 6202 genome-wide [58]) and the PAR regions are significantly enriched in sporophyte-biased genes (82 compared to 2097 genome-wide) [28,57]. Interestingly, when we plotted diversity in sliding windows and compared it with the position of these generation-biased genes, the pattern of neutral diversity on the PAR was significantly correlated with the distribution of the generation-biased genes in the windows of analysis (Figure 3A) (Kendall rank correlation test: tau = 0.18, *p* = 0.02). For Tajima’s *D* per window (Figure 3B), the correlation was not significant (Kendall rank correlation test: tau = −0.03, *p* = 0.7).

Based on the linkage map, the average recombination rate for the PAR was 19.2 cM/Mb. Four autosomes of similar size (chr4, chr5, chr21 and chr26) had average recombination levels in a similar range (between 17 and 20.6 cM/Mb). As shown before for *Ectocarpus* 7 [28], the PAR of *E. siliculosus* did not exhibit a significantly higher recombination rate on average than the autosomes. To investigate the possibility of a direct link between recombination rate and nucleotide diversity, we plotted recombination rate together with neutral diversity along the sex chromosome as well as for one representative autosome with a similar number of sliding windows for the π estimates (Figure S5). We also analyzed the correlation between neutral diversity and the recombination rate for those two chromosomes (Figure S6). In the sex chromosome, recombination rate was lower around the position of the SDR (as expected) and at the one of the telomeres (Figure S5). Correlation analysis indicated a weak negative correlation (r = −0.18) between recombination rate and nucleotide diversity, which was barely significant (*p* = 0.049). In the autosome, we observed more variation in the recombination rate than in the pattern of nucleotide diversity along the chromosome but no significant correlation between nucleotide diversity and recombination rate was observed (Figures S5 and S6).

### 3.6. Genetic Diversity in a Selected Subset of Autosomal, PAR and SDR Genes

**Figure 4:**
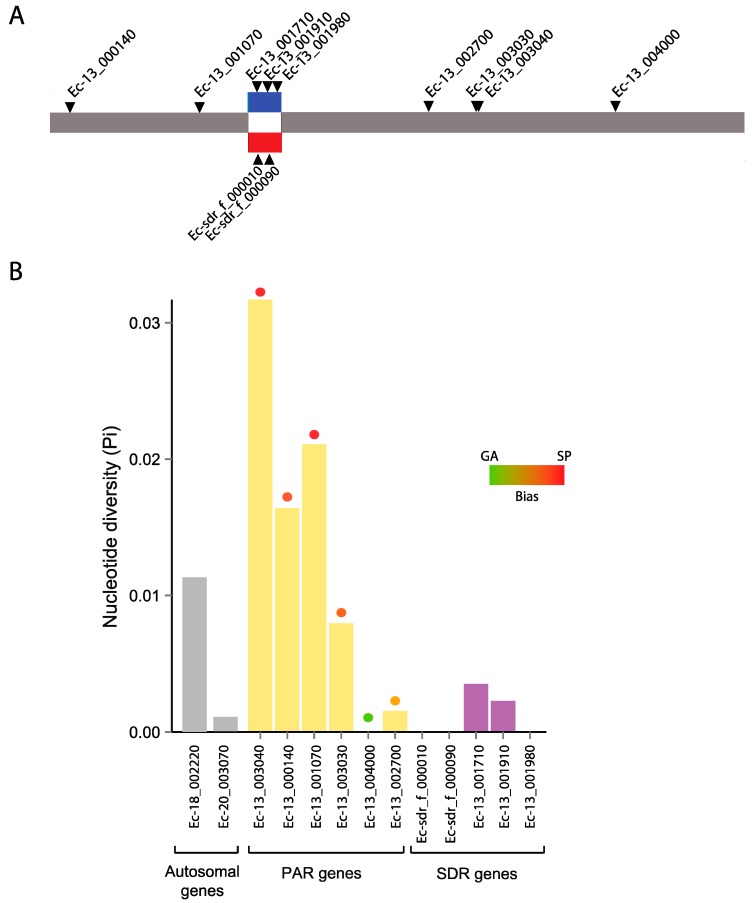
Neutral diversity of PAR and SDR genes in *Ectocarpus siliculosus*. (**A**) Physical position of the studied PAR genes on the sex chromosome. Gene names are indicated, the sex-determining region (SDR) is marked in red (female) and blue (male); (**B**) Diversity of the studied autosomal, PAR and SDR genes at synonymous sites. The level of differential expression (fold change) of the PAR genes in the sporophyte (SP) and gametophyte (GA) generations is represented by the colored circles (green denotes strong GA-bias and red stands for strong SP-bias).

ddRAD-seq data used in this study gave a broad overview of neutral diversity (because we removed regions corresponding to exons from the data before analysis) but did not provide information specifically for genes. To investigate diversity patterns at the gene level in the PAR and SDR, and in particular to test if the signal of high values of π in the PAR measured using the ddRAD-seq data was due to footprints of balancing selection on generation-biased genes, we determined π for a subset of six single-copy PAR genes and five single-copy SDR genes (two female-specific and three male-specific, Figure 4A) to study. For the PAR subset, we chose four generation-biased genes and two genes without significant bias in expression. We also sequenced two single-copy autosomal genes.

All 13 autosomal, PAR and SDR genes were successfully amplified from at least eight *E. siliculosus* individuals and aligned to the reference sequence from *Ectocarpus* 7 [52]. Nucleotide diversity statistics for all genes were studied based on synonymous sites (Table 1 and Table 2 and Figure 4).

Table 2 and Figure 4 summarize the diversity statistics for all genes, based on synonymous sites. Consistent with our ddRAD-seq data, PAR genes tended to exhibit high neutral diversity with a mean and standard error of π = 0.013 ± 0.0046, whereas SDR genes exhibited the lowest diversity (0.0012 ± 0.0007), though it should be noted that this difference was not found to be significant by a pairwise Wilcoxon test (*p* = 0.28). The two autosomal genes showed an average π = 0.0062. Consistent with the ddRAD-seq data, no obvious correlation could be observed between the neutral diversity of PAR genes in relation to their distance from the SDR (Figure 4), i.e., the genes with the highest diversity were not necessarily located closer to the SDR border. We noted, however, that all five sporophyte-biased genes showed higher π values, whereas the two genes that were not SP-biased showed lower π (Figure 4). Note however, that statistical power was low for these analyses because of the limited number of genes studied.

### 3.7. Evolutionary Histories of the Selected Autosomal, PAR and SDR Genes

Most of the seven selected PAR and SDR genes that were polymorphic exhibited negative Tajima’s *D* values, except for one gene in the PAR (Ec-13_000140) and two genes in the male SDR (Ec-13_001710 and Ec-13_001910) (Table 2). The positive DoS values for the latter two genes suggest that they are evolving under positive selection. However, a scenario of random differences being fixed due to the smaller effective population size (N_e_) of the SDR (compared to the autosomes) cannot be ruled out [59]. Out of the six PAR genes, only Ec-13_001070 appeared to be evolving neutrally. Ec-13_004000 might also be evolving neutrally but the relatively high *D*_N_/*D*_S_ value of 0.312 suggests positive selection.

The PAR gene Ec-13_003030 is also most likely evolving under positive selection (or has undergone a selective sweep) given the positive DoS value, and strongly negative Tajima’s *D* and Fay and Wu’s *H* values. Similarly, a positive DoS value suggests that Ec-13_000140 is also under adaptive selection. However, Ec-13_000140 also has a significantly positive Tajima’s *D* (*p* < 0.01) which strongly suggests that the gene may be under balancing selection, a phenomenon that cannot be detected by the DoS statistic. Please note that on the other hand, Ec-13_003040 and Ec-13_002700 seem to be evolving under relaxed purifying selection, as they have much higher polymorphism than divergence (indicated by the negative DoS value). Concerning the autosomal genes, one lacked replacements sites and hence *D*_N_/*D*_S_ could not be estimated. The second one showed a low *D*_N_/*D*_S_ value and none of them showed a significant Tajima’s *D* value.

## 4. Discussion

### 4.1. Non-Recombining Regions in U and V Chromosomes Exhibit Reduced Neutral Diversity

Reduced neutral diversity is a hallmark of Y chromosomes [60] and other non-recombining chromosomes [61]. Reduction in neutral diversity is expected to be proportional to the effective population size of the non-recombining chromosomes compared to the autosomes, leading to a prediction of ¼ as much neutral diversity on the Y chromosome compared to the autosomes in XY systems or ½ as much neutral diversity on the V or U SDRs compared to the autosomes in UV sex-determination systems, assuming a 1:1 sex ratio and similar levels of reproductive success in males and females. The data presented in this study was in line with this prediction, with the neutral diversity of the combined U and V sex-determining regions being 0.68 of the autosomal chromosomes. 

In several species, the observed levels of π for the SDR have been lower than would have been predicted based solely on this neutral effect. In *Rumex*, for example, the low level diversity observed in the Y region is consistent with an important role of linked selection, with either effects of purifying selection alone or combined with positive selection driving loss of diversity [62]. Similarly, neutral diversity on the human Y chromosome [60] and on the W chromosome of birds [63] was drastically lower than that of autosomes (5–10 times and 8–13 times, respectively). These patterns of loss of diversity could be driven by selective sweeps due to sexual selection acting on testis-specific Y chromosome genes [64]; however, they can also occur in the absence of sexual selection as shown in the example of the flycatcher’s W chromosome [63].

In contrast, the agreement between the theoretical prediction and the observed level of neutral diversity for *Ectocarpus* suggests that evolution of its SDR is not significantly driven by background selection or selective sweeps. In other words, since the observed pattern of diversity could be explained by neutral processes (e.g., stochastic processes caused by genetic drift), it is not necessary to propose additional evolutionary forces such as background selection or selective sweeps. This finding is congruent with our previous studies that failed to detect signatures of positive selection acting on a set of SDR genes that have been conserved across several brown algal species [29]. Please note that the *Ectocarpus* SDR is rather small with only 20 and 22 genes being sex-linked on the V and U chromosomes, respectively [26] and this species displays limited levels of sexual dimorphism [57]. In the absence of recombination, Hill-Robertson interference should decrease the local N_e_ and thereby diversity due to effects of linked selection, but the magnitude of this effect will depend on the number of linked selected sites [62,65,66]. Therefore, a small SDR may be less affected by Hill-Robertson interference than an SDR with many genes where there is a larger scope for selection.

### 4.2. Increased Nucleotide Diversity on the PAR Compared with Autosomes

Our data indicate that the PAR of the sex chromosome had higher median neutral diversity than any of the autosomes. Given that the PAR is also enriched in sporophyte-biased genes, one interesting possibility is that the two phenomena are connected. Such an association is supported by the observation that all of the five genes that exhibited SP-biased expression in our gene-by-gene analysis also presented high π_syn_ values and there was evidence that at least one of the genes was evolving under balancing selection.

Two of the five SP-biased genes (Ec-13_002700 and Ec-13_003040) also presented sex-biased gene expression and, overall, the PAR has been shown to be enriched in female-biased genes [28]. These observations open up the possibility that polymorphism may be maintained both by generation-antagonistic selection and by sexually-antagonistic selection, as has been proposed for *Silene* [19]. A recently proposed model to explain the spread of generation-biased alleles in the PAR of *Ectocarpus* [28] assumes that the evolution of the PAR in haploid systems is influenced by differential selection pressures in males and females acting on alleles that are advantageous during the sporophyte generation of the life cycle. One consequence of this model is that loci on the PAR that are subject to sexually-antagonistic selection would tend to be more polymorphic.

We have also considered alternative explanations for the elevated level of neutral diversity on the PAR. For example, we cannot exclude an effect of recombination rate on the variation of the nucleotide diversity [47,67,68]. Our analysis showed a weak negative correlation between recombination rate and neutral diversity, but this correlation was barely significant. No significant correlation was observed for a representative autosome. A positive correlation between DNA diversity and recombination rate has been found in many organisms including animals [69,70,71,72,73], plants [74,75,76] and fungi [77]. Although such patterns have been attributed to the action of natural selection, neutral explanations for an observed positive correlation between recombination and diversity have also been proposed, when, in addition to intraspecific variation, recombination rate was correlated with divergence (e.g., [78]). There are also cases where such a correlation has not been found. For example, in maize, single nucleotide polymorphism and recombination rate were found to be uncorrelated [79,80,81]. Additional studies will be required to depict more precisely the relationship between neutral diversity and recombination rate in *Ectocarpus* genomes.

Finally, the higher diversity of the PAR could also potentially be explained by a higher mutation rate. Elevated local mutation rate (as *D*_S_) was a strong predictor of intraspecific diversity in *Heliconius* species [47] but did not explain higher levels of π in the PAR of *Silene* [19]. In *Ectocarpus*, PAR genes have been shown to have significantly higher *D*_S_ than autosomal genes [28], so an analysis of mutation rates across the genome would be valuable to understand the role of this parameter in driving higher genetic diversity across the PAR. A thorough comparison of nucleotide variations between parents and progeny of a mapping population for instance would allow the mutation rate within the different chromosomal regions to be evaluated. Such an analysis has been carried out for the collared flycatcher [82].

### 4.3. Lack of a Regional Pattern of PAR Diversity in Relation to the SDR

Neutral sites very closely linked to any balanced polymorphism are expected to have higher diversity than surrounding genome regions [83]. Neutral diversity is thus expected to be elevated in the regions of the PAR that are closely linked to the SDR [17]. However, recombination during each generation will break down the association and the effect on diversity will become negligible unless both the recombination rate and the effective population size are small. This prediction is consistent with the observation that the peaks of polymorphism near sites known to be under long-term balancing selection are often confined to the gene itself. Therefore, under the neutral null hypothesis, only PAR genes very closely linked to the SDR should be affected [17]. Both ddRAD-seq and gene-by-gene analysis failed to find evidence for a pattern of elevated π close to the SDR border in *Ectocarpus*. The failure to find such a pattern could be due to the low resolution of ddRAD-seq markers or may be explained by a disruption of the PAR-SDR association due to gene movement and chromosomal rearrangements. The latter explanation was proposed to explain neutral diversity patterns in *Silene* [8]. Consistent with this, we showed recently that brown algal UV sex chromosomes are very dynamic with a lot of gene trafficking and rearrangements taking place [29]. Another possibility, again also suggested for *Silene* [8], is that the PAR used to be a non-recombining region, and recombination started recently. Under this hypothesis, the increased number of variants we found on the PAR could reflect a long evolutionary history of complete sex linkage. This hypothesis would also explain the intermediate characteristics of the PAR (between the autosome and SDR) in terms of GC content, TE content, gene density, *D*_S_, gene size etc. [28]. Additional information about the PARs of more brown algal species is required to fully understand the evolutionary history of these interesting chromosomal regions.

## 5. Conclusions

We have used ddRAD-seq and gene-by-gene analyses to investigate, for the first time, patterns of neutral diversity in a UV sex determination system. Our results confirm theoretical predictions for UV SDRs, which predict that the level of genetic diversity in such regions should be about half that of the autosomes. This correlation between the theoretical prediction and experimental measurements suggests that the evolution of the SDR in *Ectocarpus* is not significantly impacted by Hill-Robertson effects.

In contrast, the PAR exhibited a higher level of diversity than the autosomes and high levels of Tajima’s D, suggesting balancing selection. This observation is interesting because the PAR of *Ectocarpus* has been shown to have an unusual structure with low gene density, high transposable element content and an enrichment in both generation biased and sex-biased genes [28,57]. More work is needed to understand whether, and to what extent, these structural features underlie the high level of genetic diversity.

## Figures and Tables

**Table 1:** Selected autosomal, pseudoautosomal regions (PAR) and sex-determining region (SDR) genes analyzed in this study. In brackets after the length of the studied region, is the total length of the coding sequence.

Gene	Functional Description	No. of Sequences	Length of Region Studied (Total CDS)	Segregating Sites	No. of Haplotypes
***Autosomes***					
Ec-18_002220	Alpha tubulin	19	141 (1362)	1	2
Ec-20_003070	Similar to G-protein coupled receptors	20	747 (1650)	1	2
***PAR***					
Ec-13_003040	Expressed unknown protein	16	258 (450)	28	10
Ec-13_000140	Expressed unknown protein	19	603 (1200)	8	2
Ec-13_001070	Expressed unknown protein	12	390 (1728)	6	5
Ec-13_003030	Expressed unknown protein	9	330 (648)	5	3
Ec-13_004000	Tetratricopeptide TPR_2 repeat protein	13	423 (2997)	0	1
Ec-13_002700	Expressed unknown protein	18	606 (993)	4	4
***SDR***					
Ec-sdr_f_000010	STE20-like serine/threonine kinase	8	597 (1265)	0	1
Ec-sdr_f_000090	GTPase activating protein	9	543 (2547)	0	1
Ec-13_001710	GTPase activating protein	11	627 (1944)	2	3
Ec-13_001910	STE20-like serine/threonine kinase	9	1062 (1314)	1	2
Ec-13_001980	Ubiquitin C-terminal hydrolase	11	570 (1110)	0	1

**Table 2:** Measurements used to infer the evolutionary forces acting on autosomal, PAR and SDR genes. Significant *p*-values (*p* < 0.05) for the neutrality tests and for the bias in expression between generations are shown in boldface.

Gene	π_Syn_^#^	*D*_N_/*D*_S_	DoS	Tajima’s *D*	*p*-Value	Fay and Wu’s *H*	*p*-Value	log_2_FC (SP/GA) *
***Autosomes***								
Ec-18_002220	0.01138	0	No replacements	0.417	0.368	0.257	0.196	−0.52
Ec-20_003070	0.00106	0.13	0.316	−0.592	0.501	**−1.516**	0.015	−0.78
***PAR***								
Ec-13_003040	0.032	0.512	−0.223	**−1.740**	0.0002	−3.467	0.102	**2.25**
Ec-13_000140	0.016	0.253	0.105	**2.033**	0.0007	−1.392	0.167	**6.52**
Ec-13_001070	0.02	0	0	−0.120	0.443	−0.848	0.171	**1.19**
Ec-13_003030	0.008	0.494	0.158	**−1.678**	0.013	**−4.278**	0.0073	**0.98**
Ec-13_004000	0	0.312	no polymorphism	NA	NA	NA	NA	**0.71**
Ec-13_002700	0.002	0.109	−0.265	−1.381	0.050	−0.863	0.101	−0.44
***SDR***								
Ec-sdr_f_000010	0	0	no polymorphism	NA	NA	NA	NA	−0.27
Ec-sdr_f_000090	0	0.6	no polymorphism	NA	NA	NA	NA	0.41
Ec-13_001710	0.00351	0.692	0.190	0.199	0.399	0.255	0.412	−0.53
Ec-13_001910	0.00229	0.077	0.222	1.401	0.122	−0.139	0.210	0.16
Ec-13_001980	0	2.077	no polymorphism	NA	NA	NA	NA	−0.54

* SP—sporophyte, GA—gametophyte, FC—fold change in expression. ^#^ π_Syn_ values for synonymous sites.

## Supplementary Materials

The following are available online at http://www.mdpi.com/2073-4425/9/6/286/s1, Table S1: Samples used for genetic mapping of the sex locus of *E. siliculosus*; Table S2: Natural populations used for ddRAD sequencing and ddRAD-seq data summary; Table S3: Samples used in the analysis of the population genetics statistics in coding regions; Table S4: PCR primers used for amplification of the PAR and SDR genes; Table S5: Summary statistics of the VCF files; Figure S1. Mapping of the sex-determining region in *Ectocarpus siliculosus*; Figure S2. Population genetics statistics for the three *Ectocarpus siliculosus* genomic compartments: autosomes, PAR and SDR; Figure S3. Population genetics statistics for the individual autosomes, PAR and SDR in *Ectocarpus siliculosus*; Figure S4. Comparison of neutral diversity patterns along the sex chromosome of *Ectocarpus siliculosus* between the four study populations; Figure S5. The landscape of neutral diversity (π) and recombination rates along the sex chromosome and autosomes in *Ectocarpus siliculosus*; Figure S6. Correlation between neutral diversity and recombination rate.
